# A spectral preserved model based on spectral contribution and dependence with detail injection for pansharpening

**DOI:** 10.1038/s41598-023-33574-5

**Published:** 2023-04-27

**Authors:** Lei Wu, Xunyan Jiang, Jia Peng, Guangsheng Wu, Xiaozhen Xiong

**Affiliations:** 1 College of Mathematics and Computer, Xinyu University, Xinyu, 338004 China; 2 School of Economics and Management, Xinyu University, Xinyu, 338004 China

**Keywords:** Image processing, Imaging

## Abstract

Pansharpening integrates the high spectral content of multispectral (MS) images and the fine spatial information of the corresponding panchromatic (PAN) images to produce a high spectral-spatial resolution image. Traditional pansharpening methods compensate for the spatial lack of the MS image using the PAN image details, which easily causes spectral distortion. To achieve spectral fidelity, a spectral preservation model based on spectral contribution and dempendence with detail injection for pansharpening is proposed. In the proposed model, first, an efficacy coefficient (CE) based on the spatial difference between the MS and PAN images is designed to suppress the impact of the detail injection on the spectra. Second, the spectral contribution and dependence (SCD) between the MS bands and pixels are considered to strengthen the internal adaptation of the spectra. Finally, a spectrally preserved model based on CE and SCD is designed to force the fused image fidelity in spectra when the MS image is pansharpened with the details of the PAN image. Experimental results show that the proposed model is effective.

## Introduction

A high-resolution multispectral (HRMS) image is important for the analysis, planning, utilization and management of earth resources^1^. In fact, it is difficult for most satellites to produce HRMS images due to their physical constraints^2^. However, they use two remote sensors with contradictory functions to visit the earth. One sensor provides multispectral (MS) data with coarse spatial information^3^. Another sensor offers panchromatic (PAN) data that includes fine spatial information without spectral content^4^. To meet the demands of remote sensing data applications, fusing the data derived from the aforementioned two kinds of sensors is effective. Generally, this data processing is called pansharpening^5^.

The various research communities take different paths to expand the pansharpening methods that are primarily divided into three families. One family is the component substitution (CS) cluster, where a transformation is performed to shift the MS data into different domains, and one of them is replaced by a PAN band before the reconstituted components are inversed into the original domain. The common CS methods pansharpen remote sensing data with the intensity-hue-saturation (IHS) transform^6^, principal component analysis (PCA)^7^, and the Gram–Schmidt (GS) method^8^. The fused results generated by the CS methods easily exhibit serious spectral distortion. In contrast, the second family, called multiresolution analysis (MRA), affords an HRMS image with spatial distortion because MRA methods integrate the information of remote sensing data at multiple scales formed by multiple transformations^9^. The popular MRA methods are based on the wavelet transform, such as the nonsubsampled contourlet transform (NSCT)^10^ and the nonsubsampled shearlet transform (NSST)^11^. A critical comparison among CS and MRA pansharpening algorithms can be seen in literature^12^.

Recently, the third family, called the detail injection model (DIM), based on a hybrid of CS and MRA, has played an important role in the pansharpening field. The DIM injects the details from the PAN image into the MS image to improve the resolution of the fusion image^13^. Although the reduction of the spectral-spatial distortion is easier to implement than both the CS and MRA methods, spectral heterogeneity easily appears in the fused results because the spatial enhancement influences the spectral fidelity^14^.

To overcome this problem, a spectral preservation model based on spectral contribution and dempendence with detail injection for pansharpening is proposed. A spectral recovery algorithm is designed to construct the spectral preservation model where the spectral properties, including the spectral contribution and dependence (SCD) from the pixels in the MS image and the impact of the injection details on the original spectra, are considered simultaneously. The main contributions of this paper are the following: (1) We introduce an efficacy coefficient (CE) based on the spatial difference between the MS and PAN images to suppress the impact of the spatial enhancement on the spectra. (2) We design an SCD algorithm based on the spectral contribution and dependence to strengthen the internal adaptation of the spectra. (3) We develop a spectral preservation model based on the CE and SCD to ensure the spectral fidelity of the fused image.

## Spectral preserved model

In this section, we first introduce a DIM to pansharpen a low-resolution MS (LRMS) image with the spatial geometry information of the corresponding PAN image. The introduced DIM^13^ is defined as follows:

1 $$FMS_{k} = LRMS_{k} + g_{k} \cdot *PAN^{detail} ,\quad k = 1, \ldots ,B,$$

2 $$g_{k} = corr(LRMS_{k} ,PAN)*average\left( {\frac{{std(LRMS_{k} )}}{std(PAN)}} \right),$$

where $FMS_{k}$ and $LRMS_{k}$ are the fusion image and LRMS image, respectively, $k$ is the *k*th band, $PAN$ is the PAN image, and $g_{k}$ is an injection gain, $PAN^{detail}$ is the spatial details of $PAN$, and $corr( \cdot )$, $average( \cdot )$, and $std( \cdot )$ are functions that find the correlation coefficient, the mean value, and standard deviation, respectively.

**Figure 1:**
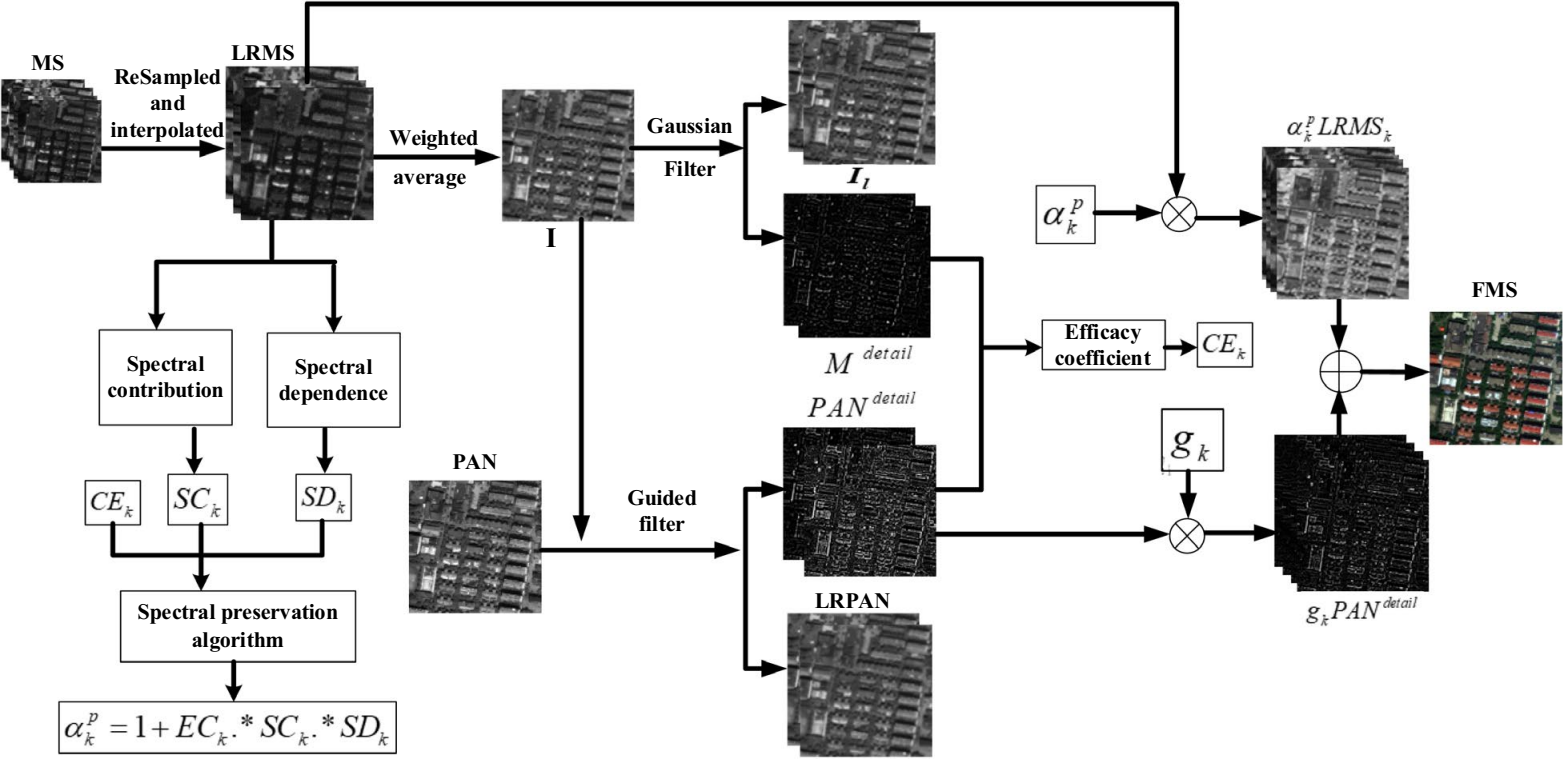
Flowchart of the proposed approach. LRPAN and $I_{l}$ are the corresponding low-pass version of the PAN and MS images after filtering the PAN and MS images two times, respectively, $M^{detail}$ and $PAN^{detail}$ are the hgh frequency details of the MS and PAN images, respectively, $SC_{k}$, $SD_{k}$ $CE_{k}$ and $a_{k}^{p}$ are the spectral contribution ratio, spectral dependent coefficient, efficacy coefficient and spectral modulation coefficient, respectively, FMS is the fused image.

Subsequently, we construct the spectral preservation model based on the introduced DIM in detail below, and the framework of the spectral preservation model is illustrated in Fig. 1.

### CE based on the extracted details

We let $LRMS_{k}$ denote the LRMS image. This is shown in Fig. 1. $LRMS_{k}$ is obtained by resampling and interpolating the MS image. The $I$ component is produced by weighting the average $LRMS_{k}$. To provide the desired details, we adopt a Gaussian filter^6^ with human visual characteristics to filter $I$ to obtain the details $M^{detail}$ because $LRMS_{k}$ has abundant color, but it has a coarse edge and texture. Meanwhile, we use the guided filter^15^ with the $I$ component as the guided image to filter the $PAN$ to obtain the details $PAN^{detail}$, because $PAN^{detail}$ in Eq. (1) needs to be highly correlated to the $LRMS_{k}$. In this study, the Gaussian filter and guided filter act on $I$ and $PAN$ two times. In the filtering operation, the input image minus the output image elucidates the details. The process of obtaining the details of $LRMS_{k}$ and $PAN$ can be seen as follows:

3 $$(I - I_{l1} ) + (I_{l1} - I_{l2} ) = M^{detail} ,$$

4 $$(PAN - LRPAN_{1} ) + (LRPAN1 - LRPAN_{2} ) = PAN^{detail} ,$$

where $I_{l1}$, $I_{l2}$, $LRPAN_{1}$ and $LRPAN_{2}$ with a lower resolution are the approximate versions of $I$ and $PAN$ at the 1 and 2 levels of the filtering operation, respectively, and $M^{detail}$ and $PAN^{detail}$ are the details from $LRMS_{k}$ and $PAN$, respectively.

According Eq. (1), $PAN^{detail}$ should be injected into $LRMS_{k}$ with the help of $g_{k}$. Thus, the original coarse edge and texture, $M^{detail}$ in $LRMS_{k}$, is enhanced. However, Zhou et al.^16^ thought $PAN^{detail}$ increases the intensity and affects edge restoration of the MS image, but that changes the hue and the saturation of the spectral value of each pixel. When only $PAN^{detail}$ is injected, the spectra of the current or neighborhood pixels closely related to $M^{detail}$ may be influenced. To suppress the impact of the spatial enhancement on the spectra, Zhou et al.^16^ proposed an efficacy coefficient (EC) based on the spatial difference of the extracted details $M^{detail}$ and $PAN^{detail}$ to modulate the spectra of $LRMS_{k}$ as follows:

5 $$EC_{k} { = }\left( {PAN^{detail} - MS_{k}^{detail} } \right)/\max \{ MS_{k} (i,j)\} ,$$

where $\max \{ \cdot \}$ is a function to define the maximum value and $(i,j)$ is the coordinate of a pixel. The performance of the $EC_{k}$ had been verified by applying it to a model of $FMS_{k} = (1 + EC_{k} ).*LRMS_{k} + PAN^{detail}$ in literature^16^.

In this paper, we introduce $EC_{k}$ into Eq. (1), the Eq. (1) can be converted into the equation as follows:

6 $$FMS_{k} = (1 + EC_{k} ) \cdot *LRMS_{k} + {\text{g}}_{k} \cdot *PAN^{detail} ,\quad k = 1, \ldots ,B$$

Thus, a prototype of the spectral preservation model is formed.

### SCD algorithm based on spectral contribution and dependent

Masi et al.^17^ confirmed that the different MS bands contain different spectral components, such as vegetation, water, and soil, and strong energy variations exist in the components associated with the spectral bands. Therefore, we think there is a spectral contribution and dependence to exist not only between the MS pixels but also between the MS bands. To achieve spectral fidelity, the optimization of Eq. (6) is not enough. It is necessary to consider the spectral contribution and dependence between the MS bands and the pixels to strengthen the internal adaptation of the spectra. In our work of literature^14^, we quantize the spectral contribution rate between the MS bands given by

7 $$SC_{k} (i,j){ = }\frac{{MS_{k} (i,j)}}{{\sum\nolimits_{k = 1}^{N} {MS_{k} (i,j)} }},$$

where $(i,j)$ is the coordinate of the pixel of row $i$ and column $j$ in an image, $SC_{k}$ is a matrix, $SC_{k} (i,j)$ is the spectral contribution rate of the pixel of row $i$ and column $j$ in the matrix $SC_{k}$. The performance of the $SC_{k}$ had been proved by applying it to a model of $FMS_{k} = (1 + EC_{k} \cdot *SC_{k} ) \cdot *LRMS_{k} + g_{k} \cdot *PAN^{detail}$ in literature^14^.

Subsquently, we model the spectral dependence between the MS pixels by finding the eigenroots of a judgment matrix constructed by pixel pairwise judgment in an MS band. Let ${\varvec{x}}_{k}$ be a column vector of the pixels of the *k*th MS band with size $M = m \times n$, which are arranged in lexicographical order. Let $x_{k}^{i}$ be the projection of $LRMS_{k} (i,j)$. After arranging each band of LRMS image into a vector in lexicographical order, we construct the judgment matrices ${\mathbf{Z}}^{k}$ of the *k*th corresponding band with the formula as follows:

8 $$z_{i,j}^{k} = \frac{{x_{k}^{j} }}{{y_{k}^{i} }}\quad s.t.\quad {\varvec{y}}_{k} = {\varvec{x}}_{k}^{T} \;and\;k = 1,2, \ldots ,K$$

where $z_{i,j}^{k}$ is the element of the coordinate position $(i,j)$ in the matrix ${\mathbf{Z}}^{k}$. Mathematically, the matrix ${\mathbf{Z}}^{k}$ can be expressed as follows:

9 $${\mathbf{Z}}^{k} { = }\left[ {\begin{array}{*{20}l} {z_{1,1}^{k} } \hfill & {\quad z_{1,2}^{k} } \hfill & {\quad \cdots } \hfill & {\quad z_{1,M}^{k} } \hfill \\ {z_{2,1}^{k} } \hfill & {\quad z_{2,2}^{k} } \hfill & {\quad \cdots } \hfill & {\quad z_{2,M}^{k} } \hfill \\ \cdots \hfill & {\quad \cdots } \hfill & {\quad \cdots } \hfill & {\quad \cdots } \hfill \\ {z_{M,1}^{k} } \hfill & {\quad z_{M,2}^{k} } \hfill & {\quad \cdots } \hfill & {\quad z_{M,M}^{k} } \hfill \\ \end{array} } \right],$$

where $M = m \times n$. We solve ${\mathbf{Z}}^{k}$ to generate the eigenvectors ${\varvec{w}}^{k}$. First, the columns of ${\mathbf{Z}}^{k}$ are normalized to obtain ${\mathbf{A}}_{{}}^{k} = (a_{ij}^{k} )_{M \times M}$, where $a_{i,j}^{k} = {{z_{i,j}^{k} } \mathord{\left/ {\vphantom {{z_{i,j}^{k} } {\sum\nolimits_{i = 1,j = 1}^{M} {z_{i,j}^{k} } }}} \right. \kern-0pt} {\sum\nolimits_{i = 1,j = 1}^{M} {z_{i,j}^{k} } }}$. Next, the rows of ${\mathbf{A}}_{{}}^{k}$ are added to generate ${\varvec{b}}^{k} = (b_{1}^{k} ,b_{2}^{k} , \ldots ,b_{M}^{k} )^{T}$, where $b_{i}^{k} = \sum\nolimits_{j = 1}^{M} {a_{i,j}^{k} }$. Third, ${\varvec{b}}^{k}$ is normalized to generate ${\varvec{c}}^{k} = (c_{1}^{k} ,c_{2}^{k} ,...,c_{M}^{k} )^{T}$, where $c_{i}^{k} = {{b_{i}^{k} } \mathord{\left/ {\vphantom {{b_{i}^{k} } {\sum\nolimits_{i = 1}^{M} {b_{i}^{k} } }}} \right. \kern-0pt} {\sum\nolimits_{i = 1}^{M} {b_{i}^{k} } }}$. Finally, we obtain ${\varvec{w}}^{k} = (c_{1}^{k} ,c_{2}^{k} ,...,c_{M}^{k} )^{T}$ and rearrange ${\varvec{w}}^{k}$ to construct a matrix $SD_{k}$ with size $r \times c$ as follows:

10 $$SD_{k}^{{}} (i,j) = w_{(i - 1)m + j}^{k} ,$$

As a result, the spectral preservation algorithm can be defined as follows:

11 $$\alpha_{k}^{p} = 1 + EC_{k} \cdot *SC_{k} \cdot *SD_{k} ,$$

where $\alpha_{k}^{p}$ is a spectral modulation coefficient.

The proposed spectral preservation model can be described as follows:

12 $$FMS_{k} = \alpha_{k}^{p} \cdot *LRMS_{k} + {\text{g}}_{k} \cdot *PAN^{detail} \; = (1 + EC_{k} .*SC_{k} .*SD_{k} ) \cdot *LRMS_{k} + {\text{g}}_{k} \cdot *PAN^{detail} ,$$

A high-quality fusion image can be provided by using the Eq. (12).

## Experimental results and analysis

### Experimental setup

**Figure 2:**
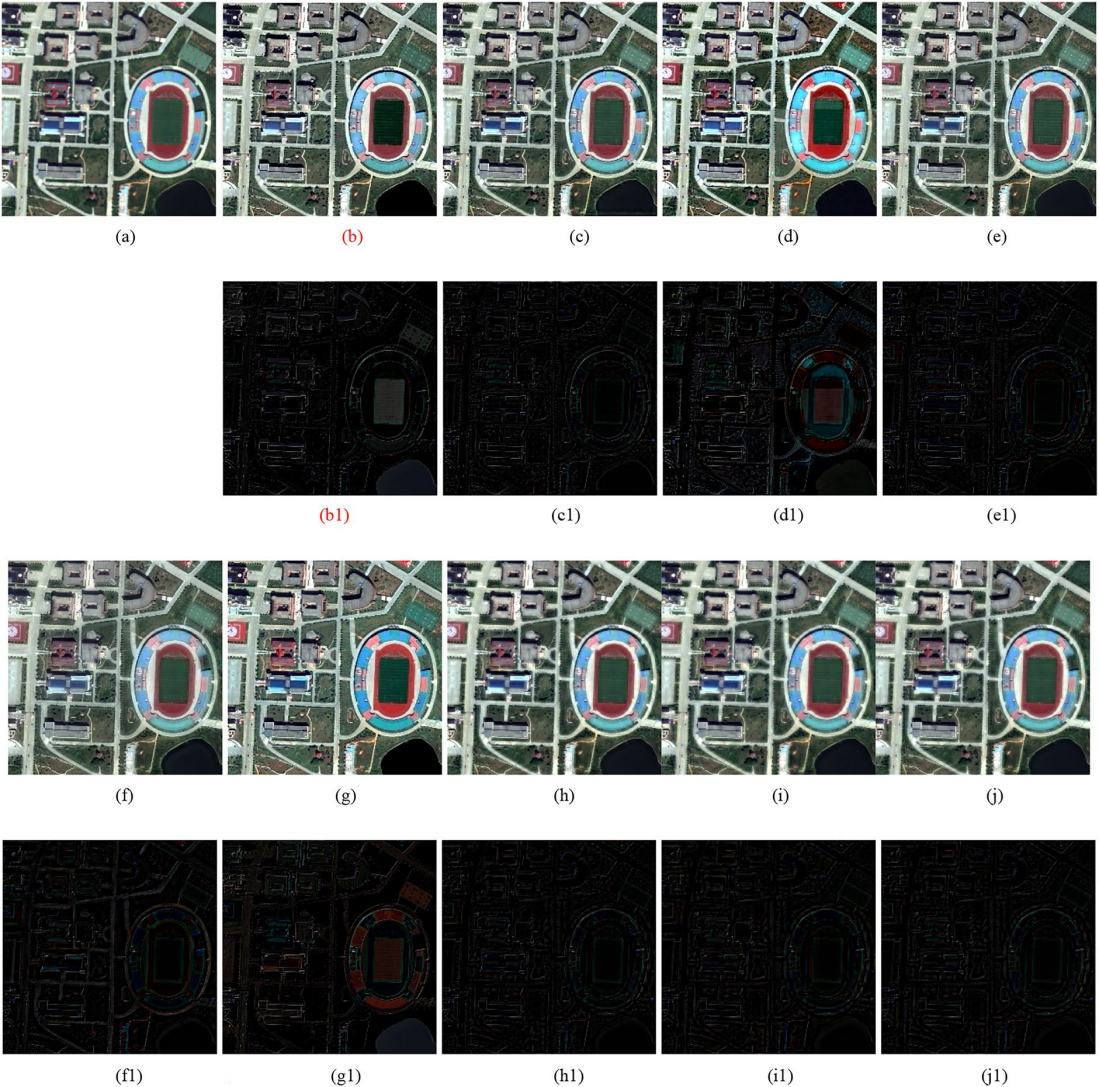
WorldView-2 images fusion results. (**a**) Ground truth. (**b**) GSA method. (**c**) CBD method. (**d**) BDSD method. (**e**) BF method. (**f**) MMMT method. (**g**) RBDSD method. (**h**) ASIM method. (**i**) DIM method. (**j**) Proposed method. (**b1**)–(**j1**) are the residual images.

**Figure 3:**
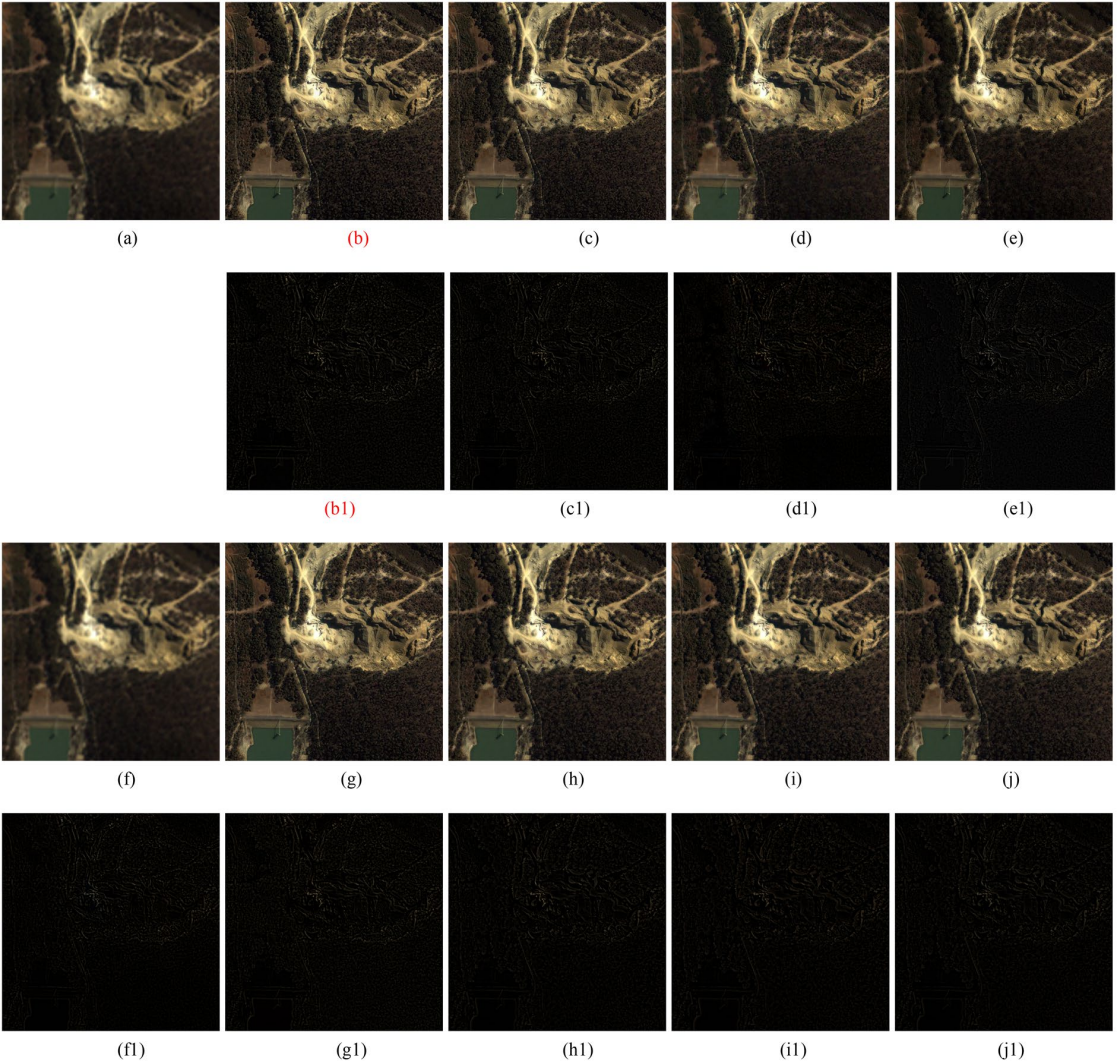
IKONOS images fusion results. (**a**) Ground truth. (**b**) GSA method. (**c**) CBD method. (**d**) BDSD method. (**e**) BF method. (**f**) MMMT method. (**g**) RBDSD method. (**h**) ASIM method. (**i**) DIM method. (**j**) Proposed method. (**b1**)-(**j1**) are the residual images.

**Figure 4:**
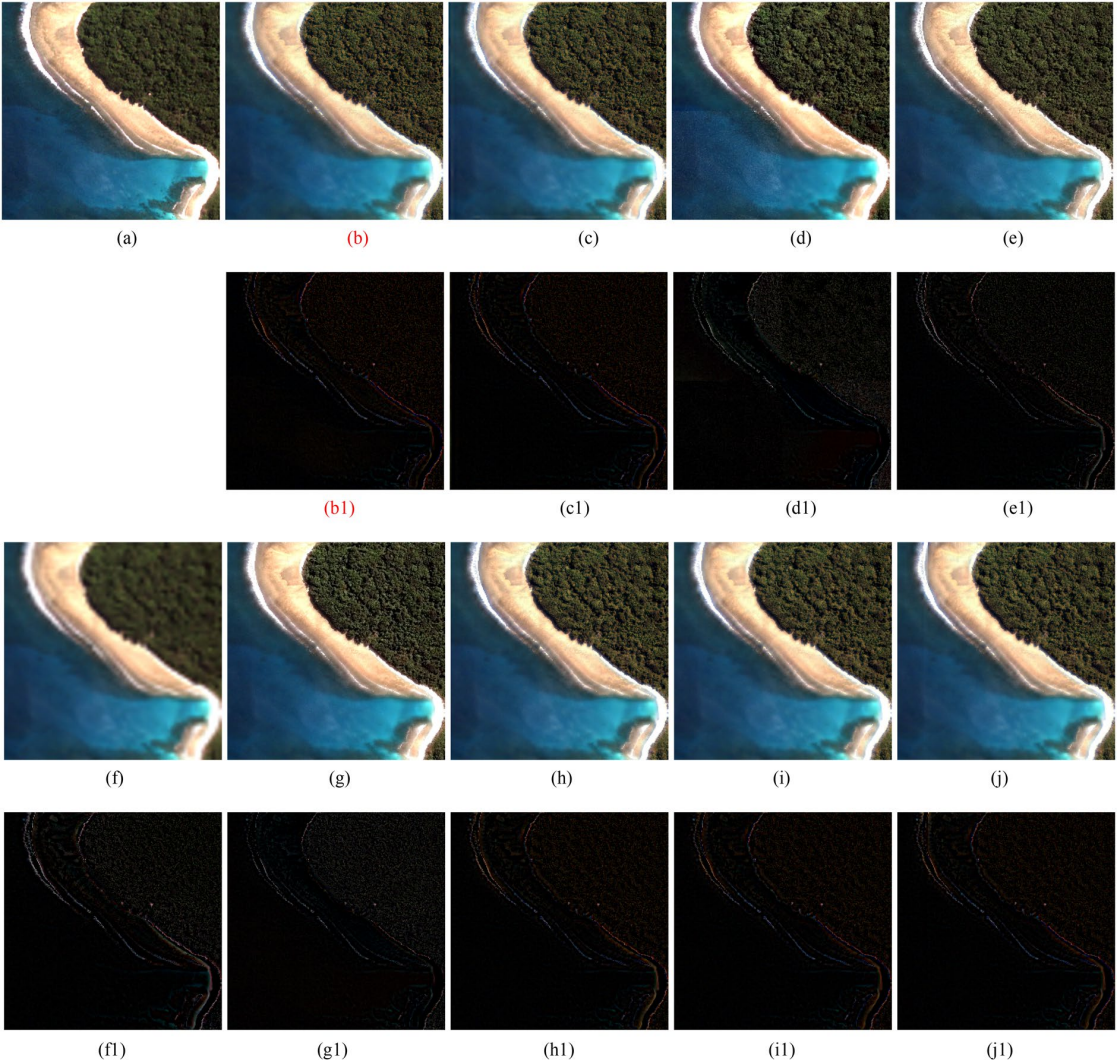
QuickBird images fusion results. (**a**) Ground truth. (**b**) GSA method. (**c**) CBD method. (**d**) BDSD method. (**e**) BF method. (**f**) MMMT method. (**g**) RBDSD method. (**h**) ASIM method. (**i**) DIM method. (**j**) Proposed method. (**b1**)-(**j1**) are the residual images.

**Figure 5:**
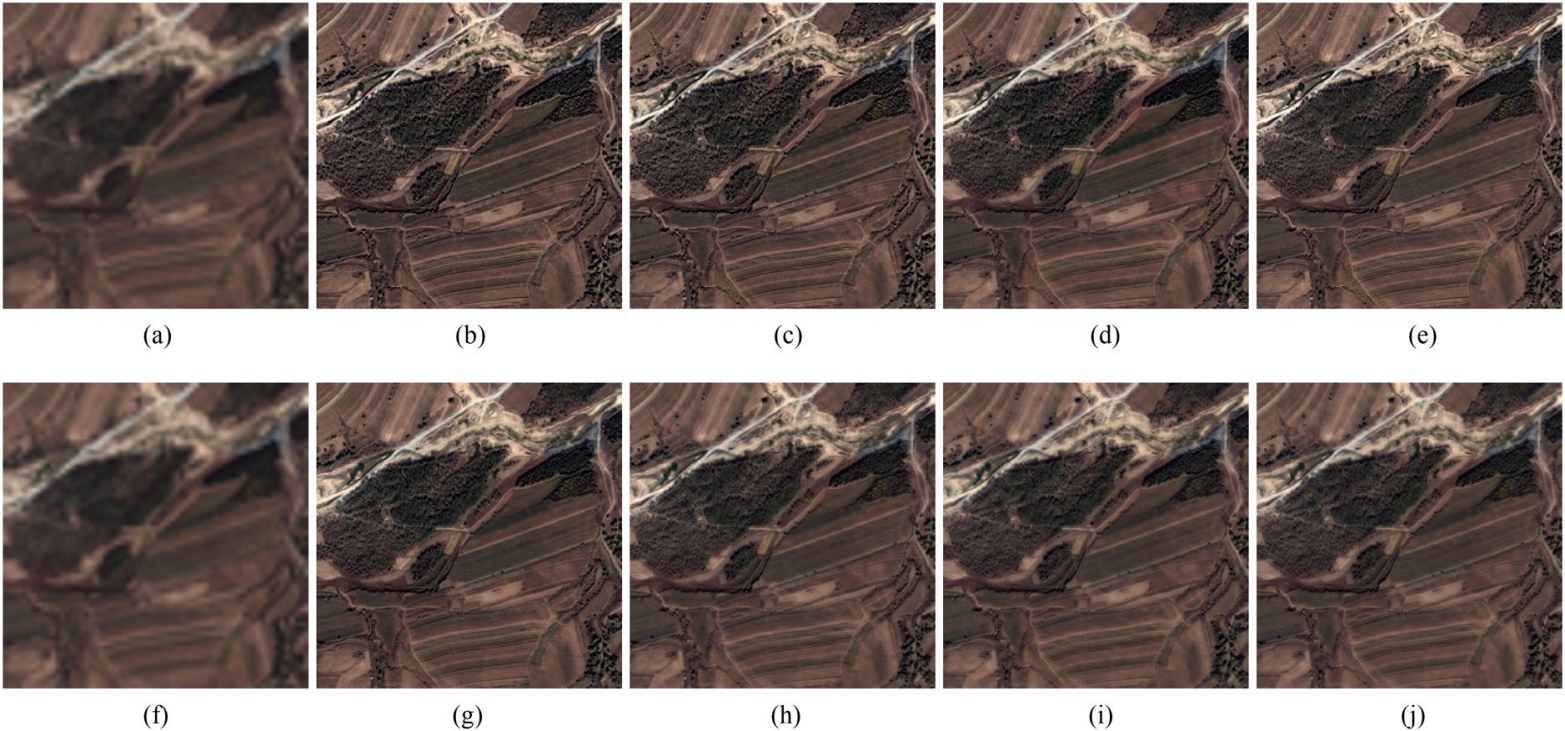
IKONOS images fusion results. (**a**) the LRMS image. (**b**) GSA method. (**c**) CBD method. (**d**) BDSD method. (**e**) BF method. (**f**) MMMT method. (**g**) RBDSD method. (**h**) ASIM method. (**i**) DIM method. (**j**) Proposed method.

**Figure 6:**
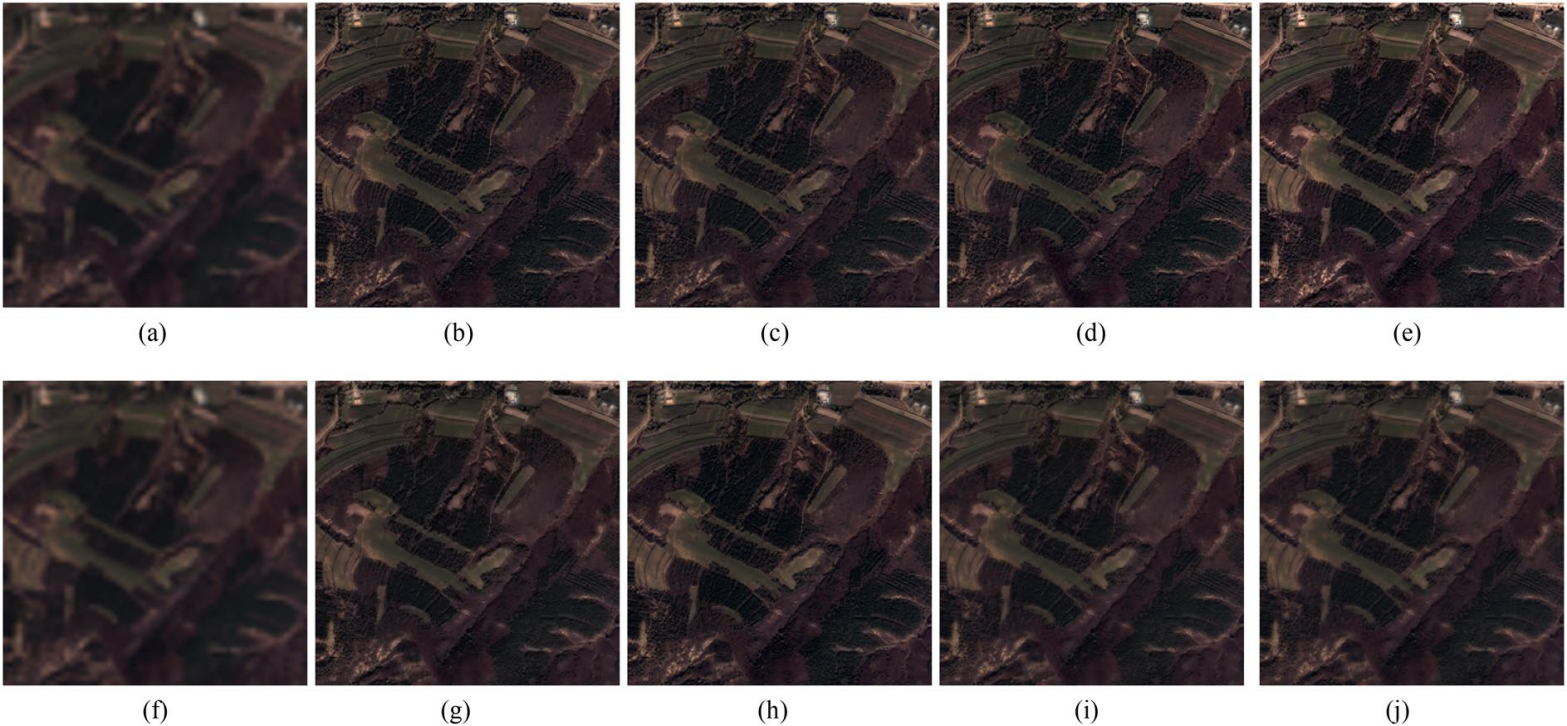
IKONOS images fusion results (**a**) the low-resolution MS image. (**b**) GSA method. (**c**) CBD method. (**d**) BDSD method. (**e**) BF method. (**f**) MMMT method. (**g**) RBDSD method. (**h**) ASIM method. (**i**) DIM method. (**j**) Proposed method.

Two types of experiments, including reduced-scale and full-scale experiments, are conducted on WorldView-2^18^, IKONOS^19^, and QuickBird^20^ datasets. The QuickBird, WorldView-2 and QuickBird satellites provides PAN images of 0.7-m, 0.5-m and 0.82-m resolution, respectively, while provides MS images of 2.8-m, 2-m and 3.28-m resolution, respectively. In order to quantitatively assess the quality of fusion results, we register and segment the images with a software of Environment for Visualizing Images and its version of 5.0 (ENVI 5.0)^27^. Since a reference image cannot be obtained in real applications, we filter and downsample the original PAN and MS images with a factor of 4 following by Wald’s protocol^28^ to obtain the reduced-scale PAN and MS images used in the reduced-scale experiments. The original MS image is regarded as the reference image. Thus, for Figs. 1, 2, 4, 5 and 6, the size of the LRMS images is 64 × 64, and the size of the corresponding reference images and PAN images of Figs. 4, Fig. 5 are all 256 × 256. For Fig. 3, the size of the LRMS image is 128 × 128, and the size of the corresponding reference image and PAN image is 512 × 512. Especially, we upsample (4 × 4) and interpolate the LRMS image to the scale of the PAN image before fusion. Two types of quantitative metrics, including with reference and without reference, are shown in Table 1. Eight methods shown in Table 2 are compared with the proposed method.

**Table 1:** The quantitative metric.

With reference image	No reference image^11^
Correlation Coefficient (CC)^13^	QNR is composed of $D_{\lambda }$ and $D_{S}$
Universal Image Quality Indices (UIQI)^13^	
Root Mean Square Error (RMSE)^13^	
Relative Average Spectral Error (RASE)^13^	$D_{\lambda }$ is the spectral distortion index
Spectral Angle Mapper (SAM)^24^	
Erreur Relative Global Adimensionnelle De Synthese (ERGAS)^13^	
The peak signal-to-noise ratio (PSNR)^25^	$D_{S}$ is the spatial distortion index
Q2n-index (Q4)^25^

**Table 2:** Compared methods.

Methods	Description
GSA	Adaptive Gram-Schmidt (GSA)^26^
CBD	The method based on context-based decision (CBD)^21^
BDSD	Band-dependent spatial detail (BDSD) injection^22^
RBDSD	Robust band-dependent spatial-detail (RBDSD)^23^
MMMT	Matting model and multiscale transform (MMMT)^11^
BF	The bilateral filtering-based (BF) method^24^
DIM	The method based on dual-injection model (DIM)^25^
ASIM	Adaptive spectral-intensity modulation (ASIM)^14^

Remark: All of the comparison methods were opened source codes offered by the corresponding authors.

### Experimental results

The results of the reduced-scale and full-scale experiments are shown in Figs. 2, 3, 4, 5 and 6. Figures 2a, 3a and 4a are the reference images and Figs. 5a, 6a are the LRMS images, respectively. Figures 2, 3, 4, 5 and 6b–j are the fused images afforded by the various methods. The results valued by the quantitative metrics are shown in Tables 3, 4, 5 and 6, where the bold black data denotes the best results. Specifically, to more accurately distinguish the difference of the fused images visually, the residual images shown in Figs. 2, 3 and 4b1–j1 are made by subtracting the reference image and the fused images.

**Table 3:** Objective evaluation of the simulation experimental results shown in Fig. 2.

Data	Method	CC↑	UIQI↑	RASE↓	RMSE↓	SAM↓	ERGAS↓	PSNR↑	Q4↑
	GSA	0.9296	0.8376	21.3716	23.3800	7.9081	5.1408	20.7539	0.8593
	CBD	0.9483	0.8551	17.1163	18.7248	5.5476	4.2108	22.6825	0.8897
	BDSD	0.9173	0.8434	24.6314	26.9462	8.5874	6.0113	19.5208	0.8438
Figure 2 (WorldView-2)	BF	0.9190	0.8509	20.8499	22.8093	5.3035	5.1375	21.4795	0.8585
	MMMT	0.9359	0.8720	18.0580	19.7550	6.0423	4.5102	22.2173	0.8315
	RBDSD	0.9193	0.8350	21.8532	23.9069	7.3277	5.2420	20.5603	0.8491
	ASIM	0.9576	0.8619	14.9121	16.3136	5.0454	3.7126	23.8798	0.8975
	DIM	0.9576	0.8622	14.7068	16.0889	5.0834	3.6743	24.0003	0.8981
	Proposed	**0.9582**	**0.8629**	**14.5789**	**15.9490**	**4.9065**	**3.6442**	**24.0761**	**0.9001**

Significant values are in [bold].

**Table 4:** Objective evaluation of the simulation experimental results shown in Fig. 3.

Data	Method	CC↑	UIQI↑	RASE↓	RMSE↓	SAM↓	ERGAS↓	PSNR↑	Q4↑
	GSA	0.9240	0.9319	25.9359	17.1587	5.2131	7.1825	23.4411	0.6167
	CBD	0.9341	0.9412	23.6292	15.6326	4.5614	6.5300	24.2502	0.6467
	BDSD	0.9268	0.9346	26.0621	17.2422	3.4231	6.2117	23.3989	0.6661
Figure 3 (QuickBird)	BF	0.9253	0.9273	28.2745	18.7058	2.7891	7.1375	22.6913	0.6348
	MMMT	0.9395	0.9488	22.0843	14.6106	**2.6746**	5.5864	24.8375	0.6753
	RBDSD	0.9268	0.9340	25.9346	17.1578	3.7974	6.4921	23.4416	0.6220
	ASIM	0.9465	0.9490	21.9868	14.5460	3.2758	5.6817	24.8759	0.6962
	DIM	**0.9544**	0.9557	20.2419	13.3917	3.5851	5.3488	25.5786	0.7164
	Proposed	**0.9544**	**0.9564**	**19.9736**	**13.2142**	3.4380	**5.2964**	**25.7100**	**0.7181**

Significant values are in [bold].

**Table 5:** Objective evaluation of the simulation experimental results shown in Fig. 4.

Data	Method	CC↑	UIQI↑	RASE↓	RMSE↓	SAM↓	ERGAS↓	PSNR↑	Q4↑
	GSA	0.9717	**0.8644**	19.1521	15.4749	5.4770	5.5985	24.3383	0.7713
	CBD	0.9740	0.8619	18.3254	14.8069	5.3204	5.1915	24.7215	0.8011
	BDSD	0.9694	0.8568	19.8281	16.0211	5.6782	5.6564	24.0369	0.7370
Figure 4 (Pleiades)	BF	0.9615	0.8481	21.4739	17.3509	**2.9446**	6.7633	23.3444	0.7663
	MMMT	0.9629	0.8441	21.4364	17.3206	5.5807	5.6090	23.3595	0.7651
	RBDSD	0.9733	0.8606	18.4376	14.8976	6.1645	4.9305	24.6685	0.7727
	ASIM	0.9773	0.8624	17.3565	14.0241	5.1074	4.7221	25.1933	0.8123
	DIM	0.9776	0.9795	17.0515	13.7776	5.1684	4.7211	25.3473	0.8158
	Proposed	**0.9786**	0.8628	**16.7308**	**13.5185**	5.0738	**4.5180**	**25.5122**	**0.8161**

Significant values are in [bold].

**Table 6:** Objective evaluation of the results of Figs. 5, 6.

Figure 5 (IKONOS)				Figure 6 (IKONOS)		
Method	$D_{\lambda }$↓	$D_{s}$↓	QNR↑	$D_{\lambda }$↓	$D_{s}$↓	QNR↑
GS	0.0627	0.1630	0.7845	0.0683	0.1877	0.7568
CBD	0.0536	0.1528	0.8018	0.0656	0.1760	0.7700
BDSD	0.0407	0.1355	0.8293	**0.0145**	0.1628	0.8250
BF	0.0784	0.1614	0.7729	0.0323	0.2328	0.7432
MMMT	0.0380	0.1206	0.8460	0.0570	0.1314	0.8191
RBDSD	0.0479	0.1341	0.8244	0.0322	0.2018	0.7725
ASIM	0.0501	0.1284	0.8280	0.0406	0.1392	0.8259
DIM	0.0384	0.1164	0.8497	0.0413	0.1314	0.8327
Proposed	**0.0335**	**0.1134**	**0.8569**	0.0335	**0.1300**	**0.8365**

Significant values are in [bold].

Subjectively, there is a substantial feature difference between the various satellite datasets. The images in Figs. 2, 3 and 4have rich color, but the color difference is small and the tone is gentle in the images in Figs 5, 6. Experimental results confirm that our method shows excellent performance, but the compared methods have worse and more unstable performances. For example, from Figs. 2, 3, 4, 5 and 6, the results afforded by the MMMT method are blurry, and the GSA, CBD, BDSD, BF and RBDSD methods fuse the WorldView-2 and IKONOS images to result in various degrees of spectral distortion. Specifically, serious spectral distortion is caused by the GSA, BDSD and RBDSD methods in Fig. 2b,d and g. Although the fusion results of the ASIM, DIM, and the proposed methods is difficult to distinguish subjectively, the results from the residual images show that our method has the best performance, and the results from Tables 3, 4, 5 and 6 quantitatively confirm that our method outperforms all the comparison methods because the objective value of our method is the best observing the quantized values of the corresponding Figs. 2, 3, 4, 5 and 6 both with reference and without reference, except for the second in SAM index of the Fig. 3, 4 and the second in UIQI index of Fig. 4 in Tables 3, 4 and 5, and the third in the $D_{\lambda }$ index of the Fig. 6 in Table 6.

## Conclusion

In this paper, a spectral preservation model is constructed to fuse remote sensing images. The proposed model deals with pansharening with adaptive detail injection, while also enforcing spectral fidelity by implementing a spectral preservation algorithm. The proposed algorithm considers not only the impact of the injection detail on the spectra but also the spectral dependence existing between the MS pixels, and bands to reduce the spectral distortion. Two groups of images with different attributes are used in the reduced-scale and full-scale experiments. Eight compared methods and two types of popular quantitative metrics are employed to test the performance of the proposed model. The results confirm that the proposed model can effectively suppress the influence of spatial enhancement on the spectra and strengthen the internal adaptation of the spectra to ensure spectral fidelity.

## Data Availability

The data that support the findings of this study are available from the corresponding author upon reasonable request.
